# Relationship between diabetic peripheral neuropathy and adherence to the Mediterranean diet in patients with type 2 diabetes mellitus: an observational study

**DOI:** 10.1007/s40618-024-02341-2

**Published:** 2024-03-18

**Authors:** S. Zúnica-García, J. J. Blanquer-Gregori, R. Sánchez-Ortiga, M. I. Jiménez-Trujillo, E. Chicharro-Luna

**Affiliations:** 1 Department of Behavioural Sciences and Health, Nursing Area, Faculty of Medicine, Miguel Hernández University, Ctra N332, Km 87, 03550 San Juan de Alicante, Alicante, CP Spain; 2 Faculty of Medicine, Miguel Hernández University, Alicante, Spain; 3 Endocrinology and Nutrition Department, Dr. Balmis General University Hospital, Alicante, Spain; 4 Department of Medicine and Surgery, Psychology, Preventive Medicine and Public Health and Medical Microbiology and Immunology, Nursing and Stomatology, Faculty of Health Sciences, Rey Juan Carlos University, Madrid, Spain

**Keywords:** Diabetes mellitus type 2, Mediterranean diet, Peripheral neuropathy, Pressure sensitivity, Monofilament test

## Abstract

**Purpose:**

The main study goal is to assess the relationship between adherence to the mediterranean diet (MD) and the presence of diabetic peripheral neuropathy (DPN) in patients with type 2 diabetes mellitus (T2DM).

**Methods:**

Observational pilot study of 174 patients diagnosed with T2DM. Sociodemographic and anthropometric variables, physical activity, smoking habits, blood biochemical parameters and comorbidities were recorded. The presence of alterations in sensitivity to pressure, pain, thermal and vibration was explored. Good MD adherence was a score  ≥  9 the 14-point MD adherence questionnaire (MEDAS-14).

**Results:**

The study population consisted of 174 patients (61.5% men and 38.5% women), with a mean age of 69.56 ± 8.86 years; 19% of these patients adhered to the MD. The score obtained in the MEDAS-14 was higher in patients who did not present alterations in sensitivity to pressure (*p* = 0.047) or vibration (*p* = 0.021). The patients without diabetic peripheral neuropathy were more likely to comply with the MD and had a higher score on the MEDAS-14 (*p* = 0.047). However, multivariate analysis showed that only altered sensitivity to pressure was associated with adherence to the MD (altered sensitivity OR = 2.9; 95%CI 1.02–8.22; *p* = 0.045).

**Conclusions:**

Although the patients with DPN had lower scores on the MEDAS questionnaire and therefore poorer adherence to the mediterranean diet, the only parameter significantly associated with the MD was that of sensitivity to pressure (monofilament test).

## Introduction

Diabetes mellitus (DM) is a global health problem that has reached alarming levels. Its worldwide prevalence is around 10.5% and this is expected to reach 12.2% by 2045, with the number of patients affected rising from 536.6 to 738.2 million [1]. Type 2 Diabetic Mellitus (T2DM) accounts for 90–95% of all cases of diabetes [2].

Diabetic neuropathy is the most common chronic complication of DM and is the first cause of ulceration in the foot [3]. Within this condition, diabetic peripheral neuropathy (DPN) has the highest prevalence [4]. It can provoke a loss of protective, thermal and vibratory sensation, together with muscle weakness, the absence or reduction of deep tendon reflexes, and slowness in nerve conduction, with sensory involvement predominating over motor impairment. Of all the healthcare resources spent on DM, 20–40% is allocated to diabetic complications affecting the foot [5].

DPN and peripheral arterial disease increase patients’ susceptibility to ulceration, infection and gangrene. Approximately 15% of diabetics will develop a foot ulcer during the course of the disease, sometimes leading to amputation of the foot or leg. Patients with T2DM who have suffered an amputation of the lower limb present 75% greater risk of mortality than those who have not undergone any such amputation, and the risk is even greater when the amputation is performed above the knee [6].

Furthermore, ulcers impose severe economic burdens on society, both to the health system directly and also due to the loss of productivity, since diabetic foot complications are one of the main causes of disability [7, 8]. Moreover, almost half of patients with ulcers are subject to depression [9].

In recent years, there has been growing interest in determining the beneficial effects of the Mediterranean diet (MD) on patients with T2DM. This diet is characterised by the intake of foods based on traditional agriculture, such as cereals, legumes, fruits, vegetables, nuts and olive oil, together with the moderate consumption of poultry, fish, dairy products and wine and the very limited consumption of red meat and sugar. In 2010, UNESCO recognised the MD as part of the Intangible Cultural Heritage of Humanity [10].

Previous studies have found that the MD plays an important role in reducing the risk of T2DM developing [11–15], delaying the need to start antidiabetic drugs [16, 17] and even achieving remission of the disease [17]. The diet is also associated with reduced levels of Hb1Ac [17–20], reduced body weight [20, 21], reduced incidence of cardiovascular risk factors [20–22] and fewer DM-associated microvascular complications [22]. In this respect, too, the PREDIMED study demonstrated that omega-3 fatty acids from fish had anti-inflammatory effects that reduced the risk of diabetic retinopathy and metabolic syndrome in patients with DM [23].

Additionally, dietary control is paramount in reducing obesity, which has been demonstrated to be associated with respiratory and cardiometabolic diseases such as T2DM, linked to pathological remodeling of subcutaneous adipose tissue [24].

However, very little is known about how adherence to the MD influences the development of DPN in patients with T2DM, and about the sensory and motor components that may be affected in its presence. Therefore, the main aim of this study is to evaluate the relationship between adherence to the MD and the presence of DPN in patients with T2DM.

## Material and methods

This observational pilot study was designed in accordance with The Strengthening the Reporting of Observational Studies in Epidemiology (STROBE) guidelines [25]. It was carried out at an Endocrinology clinic of a hospital and at a Primary Care centre from December 2020 to July 2023. The project was approved by the corresponding Ethics Committee (reference: CEIm P12019-106).

The following inclusion criteria were applied: patients diagnosed with T2DM five or more years previously, of legal age, whose mother tongue was Spanish and who agreed to participate in the study. The exclusion criteria were life expectancy of less than 6 months; the amputation of both feet; the presence of a neuropathy not arising from diabetes; or the presence of any mental illness or cognitive alteration that prevented understanding of the questionnaire.

We calculated that a sample size of 174 (157 + 10% losses) patients would be required for the reference population, taking into account an estimated 82% prevalence of patients who do not comply with the MD [26], with a confidence level of 95%, a precision of 6% and loss to follow up of 10%. All patients were included consecutively in the study group.

### Data collection

In every case, a clinical interview was conducted to determine the sociodemographic variables (sex, age, nationality, marital status, level of education and type of family life). The history of T2DM and the patient’s age at diagnosis were also noted. Smoking habits were grouped into three categories: non-smoker, ex-smoker or smoker, together with the duration of the smoking habit (for smokers) and time elapsed since giving up (for the ex-smokers). Physical activity was assessed by querying the patient about the type of activity performed on a weekly basis (walking, running, gymnastics, swimming, cycling, tennis/paddle, and/or others), as well as the frequency in minutes. It was considered that the patient engaged in physical activity when meeting the World Health Organization (WHO) recommendations, i.e., more than 150 min per week. Weight (kg) and height (cm) were recorded using the Bamed^®^ brand stadiometer scale. The body mass index (BMI) was calculated using the following formula: weight (kg)/height^2^ (m), and each patient was then classified following the WHO criteria as: normal weight (BMI 18.50–24.99), overweight (BMI 25–29.9) or obesity (BMI ≥ 30).

The degree of metabolic control was assessed by a blood test carried out during the 6 months prior to inclusion in the study (basal blood glucose (mg/dL), HbA1c (%l), triglycerides (mg/dL), total cholesterol (mg/dL), LDL cholesterol (mg/dL) and HDL cholesterol (mg/dL)). Possible comorbidity was determined by asking the patient about the presence of any complications associated with T2DM.

Sensitivity to pressure was determined using a Semmes–Weinstein 5.07–10 g SensifilTM monofilament (Novalab Ibérica^®^) [27, 28]; sensitivity to pain was determined using a Neurotip™ 40 g (Neuropen^®^) (pin-prick test) [29]; thermal sensitivity was determined using a thermal bar; sensitivity to vibration was determined using a Rydel-Seiffer 128 Hz graduated tuning fork [30]. Sensitivity to pressure, pain and temperature was explored at 12 anatomical points: in the dorsal area of the big toe (nail matrix), in the first intermetatarsal space and at the head of the fifth metatarsal; at the plantar level, in the first, third and fifth toes, at the head of the first, third and fifth metatarsals, in the internal and external longitudinal arch and at the heel. All points examined were evaluated randomly, but avoiding applying the devices to ulcers, hyperkeratosis, scars or necrotic tissue. Each sensitivity value was considered altered when the patient was unable to detect the stimulus in at least four anatomical areas in one of the two feet.

Sensitivity to vibration was assessed on the following bony prominences: the dorsum of the big toe (interphalangeal joint), the head of the first metatarsal, the head of the fifth metatarsal, the medial malleolus and the external malleolus of the ankle, on both feet. Three measurements were taken at each anatomical area explored, and the value obtained was considered altered when the average of the measurements taken in each area was ≤ 6 in patients aged under 60 years, and ≤ 4 in patients aged over 80 years [31]. Finally, the measurements taken in the five anatomical areas on each foot were averaged to obtain a final assessment of whether the patient experienced alterations in the sensitivity considered.

Finally, the patient was considered to have DPN when both the Semmes Weinsten 5.07–10 g monofilament test and another test indicated altered values [32, 33]. The American Diabetes Association guideline recommends that the monofilament test should not be used alone to diagnose this condition [32]. When more than one test is applied, the resulting sensitivity to DPN is greater than 87% [34]. For the purposes of the present study, a patient was considered to have peripheral neuropathy when at least one foot was affected.

The degree of adherence to the MD was determined using the the 14-point MD adherence questionnaire (MEDAS-14), following its previous use in the PREDIMED study [23]. This questionnaire consists of 14 questions regarding the consumption of the main components of the MD (olive oil, nuts, fruits and vegetables, fish and legumes). The final score was obtained from the sum of the 14 questionnaire items. The patient was considered to present good adherence to the MD when this score was  ≥ 9.

### Statistical analysis

The statistical analysis was performed using the statistical software Statistical Package of Social Sciences (SPPS^®^) v.28.

The qualitative variables are described by frequency distribution (counts and percentages), and the quantitative ones by the mean and the standard deviation. A bivariate inferential analysis was carried out using the chi-square test or Fisher’s exact test for the qualitative variables, and the Mann–Whitney *U* test or the Kruskal–Wallis test and Student’s *T* test for independent samples for non-parametric and parametric quantitative variables, respectively. As the study sample consisted of more than 50 individuals, the Shapiro–Wilk method was used to determine whether a variable presented a normal (parametric) distribution. A result was considered statistically significant when the *p* value < 0.05. Logistic regression was performed to identify the factors related to adherence to the MD.

## Results

The study sample consisted of 174 patients, of whom 61.5% were men and 38.5% women, with a mean age of 69.56 ± 8.86 years (95% CI 68.23–70.88). The nationality of the sample was 97.7% Spanish, 1.1% Algerian, 0.6% French and 0.6% Cuban. The mean BMI was 29.57 ± 4.74 (95% CI 28.86–30.28), which corresponds to overweight according to the WHO classification. The mean evolution of T2DM was 15.34 ± 9.83 years (95% CI 13.87–16.81) and the mean age at diagnosis of T2DM was 54.32 ± 11.32 years (95% CI 52.63–56.02). 55.1% of the patients had no formal education qualifications or had only completed primary education.

37.9% led a sedentary life and 15.5% were smokers at the time of the study, with a mean duration of tobacco consumption of 44.75 ± 13.20 years (95% CI 39.63–49.87). 52.3% were ex-smokers, having given up smoking 19.01 ± 13.27 years previously (95% CI 16.25–21.77).

The comorbidity most commonly associated with T2DM was dyslipidaemia (82.8%), followed by arterial hypertension (77%) and cardiac insufficiency (31%). Of the metabolic control parameters considered, basal glycaemia (55.7%), Hb1Ac (42.5%) and triglycerides (35.6%) were below the therapeutic levels recommended by the American Diabetes Association (ADA).

Only 19% of the patients adhered to the MD. The mean score obtained in the MEDAS-14 was 7.05 ± 1.71 (95% CI 6.79–7.30). In the bivariate analysis, a significant relationship was observed between sex (*p* = 0.003; *p* < 0.001), level of education (*p* = 0.003; *p* < 0.001) and physical activity (*p* < 0.001; *p* = 0.003) with the MEDAS-14 score and MD adherence, respectively. A statistically significant relationship was also observed between the duration of T2DM and adherence to the MD (*p* = 0.035) (see Table 1).

**Table 1:** Sociodemographic characteristics of the sample

Sociodemographic characteristics	MEDAS-14 score (*n* = 174) mean ± SD	*P* value	Adherence to MD (score ≥ 9) *n* = 33 (%)	Non-adherence to MD (score < 9) *n* = 141 (%)	*P* value
*Sex*					
Male	6.72 ± 1.58	0.003^a,c^	9 (27.3)	98 (69.5)	< 0.001^C^
Female	7.57 ± 1.78		24 (72.7)	43 (30.5)	
*Age*					
< 65 years	6.65 ± 1.67	0.143^b^	7 (21.2)	41 (29.1)	0.645
65–75 years	7.13 ± 1.85		15 (45.5)	60 (42.6)	
> 75 years	7.31 ± 1.49		11 (33.3)	40 (28.4)	
*Nationality*					
		0.317			0.811
Spanish	7.08 ± 1.72		137 (97,2)	33 (19.4)	
Algerian	5.50 ± 0.71		2 (1.4)	0 (0)	
Cuban	6		1 (0.7)	0 (0)	
French	6		1 (0.7)	0 (0)	
*Educational level*					
No formal or primary studies	6.67 ± 1.64	0.003^a,c^	9 (27.3)	87 (61.7)	< 0.001^C^
Secondary or university studies	7.51 ± 1.71		24 (72.7)	54 (38.3)	
*Type of family life*					
		0.218^a^			0.131
Live alone	7.39 ± 2.06		9 (27.3)	22 (15.6)	
Live witg someone	6.98 ± 1.62		24 (72.7)	119 (84.4)	
*Marital status*					
Single	6.33 ± 1.51	0.577^b^	1 (3)	5 (3.5)	0.819
Married	7.02 ± 173		21 (63.6)	90 (63.8)	
Separated or divorced	7 ± 1.92		5 (15.2)	15 (10.6)	
Widowed	7.05 ± 1.71		6 (18.2)	31 (22)	
*Smoking habit*					
Current smoker	6.81 ± 1.76	0.664^b^	3 (9.1)	24 (17)	0.283
Ex-smoker	7.05 ± 1.63		16 (48.5)	75 (53.2)	
Never smoked	7.15 ± 1.83		14 (42.4)	42 (29.8)	
*Physical activity*					
Sedentary lifestyle	6.36 ± 1.67	< 0.001^a,c^	5 (15.2)	61 (43.3)	0.003^c^
Physically active	7.47 ± 1.71		28 (84.8)	80 (56.7)	
*Duration of T2DM*					
< 10 years	7.2 ± 1.82	0.244^b^	15 (45.5)	40 (28.4)	0.035^c^
10–20 years	6.84 ± 1.67		10 (30.3)	78 (55.3)	
> 20 years	7.39 ± 1.71		8 (24.2)	23 (16.3)	

^a^Mann–Whitney *U* test

^b^Kruskal–Wallis test and chi-square test

^c^*p* < 0.05 statistically significant

*T2DM* Type 2 diabetes mellitus, *MD* Mediterranean diet, *MEDAS* Mediterranean Diet Adherence Screener (score range: 0–14)

Physical activity: more than 150 min per week

A statistically significant relationship was observed between BMI and the MEDAS score (*p* = 0.047), being higher in patients with normal weight (7.56 ± 1.48) than in patients with overweight or obesity (6.72 ± 1.81 and 7.19 ± 1.64 respectively). However, no metabolic or anthropometric control parameter, as well as no comorbidity, correlated significantly with adherence to the MD (see Table 2).

**Table 2:** Parameters for metabolic, anthropometric control, and comorbidities, and their relationship with adherence to the MD

	Adherence to MD (score ≥ 9) *n* = 33 (%) mean ± SD	Non-adherence to MD (score < 9) *n* = 141 (%) mean ± SD	*P* value
*BMI* (kg/m^2^)			
Normal weight (BMI < 25)	7 (21.2)	20 (14.2)	0.312
Overweight (BMI 25–29)	10 (30.3)	62 (44)	
Obesity (BMI ≥ 30)	16 (48.5)	59 (41.8)	
*Metabolic control parameters*			
Basal blood glucose (mg/dl)	130.85 ± 6.96	138.79 ± 42.36	0.449^a^
Hb1Ac (%l)	6.96 ± 0.99	7.08 ± 1.21	0.789^a^
Triglycerides (mg/dl)	157.15 ± 75.40	148.64 ± 89.18	0.317^a^
Total cholesterol (mg/dl)	170.52 ± 37.82	163.02 ± 47.04	0.174^a^
Cholesterol HDL (mg/dl)	50.69 ± 12.33	48.05 ± 14.90	0.129^a^
Cholesterol LDL (mg/dl)	93.10 ± 29.98	85.70 ± 36.62	0.119^a^
*Comorbidities*			
Coronary heart disease	5 (15.2)	29 (20.6)	0.480
Hypertension	25 (75.8)	109 (77.3)	0.849
Cerebrovascular disease	4 (12.1)	14 (9.9)	0.751
Hearts problems	8 (24.2)	46 (32.6)	0.349
Dyslipidaemia	27 (81.8)	117 (83)	0.874
Nephropathy	11 (33.3)	41 (29.1)	0.631
Retinopathy	9 (27.3)	40 (28.4)	0.900
Cancer	8 (24.2)	28 (19.9)	0.576
Depression	8 (24.2)	34 (24.1)	0.988
Dialysis	0 (0)	3 (2.1)	1^¶^
DFU	3 (9.1)	33 (23.4)	0.068
Amputations	0 (0)	14 (9.9)	0.075^¶^
Lung diseases	8 (24.2)	44 (31.2)	0.431

^a^Mann–Whitney U test

^b^Kruskal–Wallis test and chi square test or ^¶^Fisher’s exact test

^c^*p* < 0.05 statistically significant

*MD* Mediterranean diet, *BMI* Body mass index *Hb1Ac* Glycosylated hemoglobin *DFU* Diabetic foot ulcer

Sensitivity to vibration was altered in 94.3% of patients, in at least one foot. This was the form of sensitivity most severely affected. It was followed by thermal sensitivity (89.1%), pressure (32.8%) and pain (2.3%). Overall, 31.2% of patients had DPN.

**Fig. 1:**
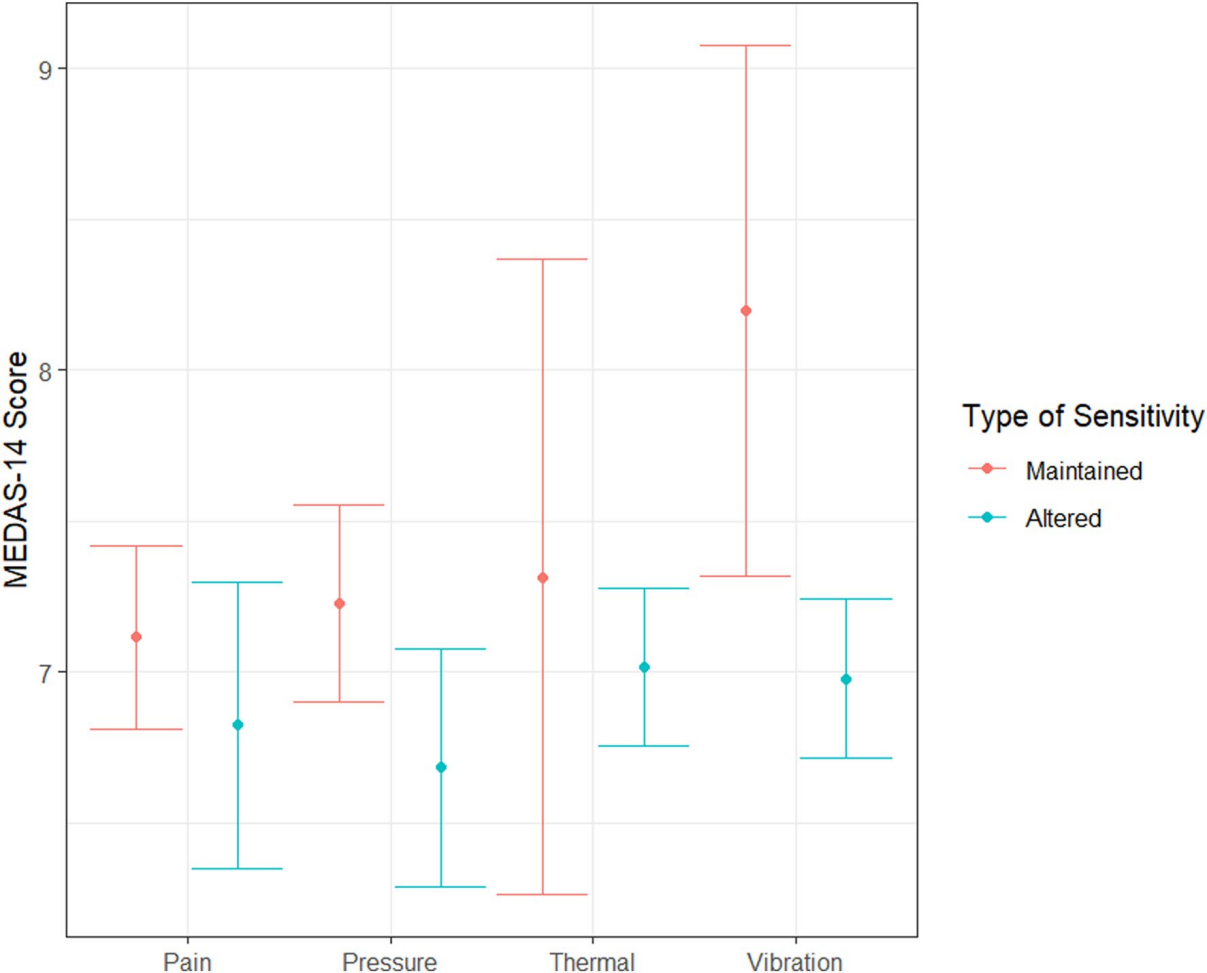
Relationship between the MEDAS-14 score and the type of sensitivity affected.

The bivariate analysis revealed a significant relationship between the MEDAS score and altered sensitivity to pressure (*p* = 0.047) and to vibration (*p* = 0.021). In both cases the score was lower (i.e., adherence was worse) among the patients affected by the loss of sensitivity. (see Fig. 1). A statistically significant inverse association was observed between pressure sensitivity and DPN and compliance with the MD (*p* = 0.017), i.e. patients who did not comply with the MD were more likely to present DPN. Corroborating this association, the mean MD score was significantly higher among the patients who did not have DPN (7.23 ± 1.79 vs 6.68 ± 1.49; *p* = 0.047).

We also analysed the relationship between the score for each questionnaire item and alterations in sensitivities, finding that the increased sensitivity to pressure, thermal and vibration was less acute among patients who consumed stir-fried vegetables at least twice a week (*p* = 0.043, *p* = 0.004 and *p* = 0.007, respectively). Similarly, increased sensitivity to pain was less acute among the patients who consumed legumes at least 3 times/week (*p* = 0.020) and among those who consumed more white than red meat (*p* = 0.020) (see Table 3).

**Table 3:** Relationship between the items of the MEDAS-14 questionnaire and the different sensitivities assessed (pressure, pain, thermal and vibration)

MEDAS-14	Category	Pressure sensitivity		P value	Pain sensitivity		P value	Thermal sensitivity		P value	Vibration sensitivity		*P* value
		Maintained *n* = 117	Altered *n* = 57		Maintained *n* = 134	Altered *n* = 40		Maintained *n* = 19	Altered *n* = 155		Maintained *n* = 10	Altered *n* = 164	
		*n* (%)	*n* (%)		*n* (%)	*n* (%)		*n* (%)	*n* (%)		*n* (%)	*n* (%)	
Question 1 Olive oil as the primary fat	Yes	108 (92.3)	51 (89.5)	0.570^¶^	124 (92.5)	35 (87.5)	0.340^¶^	17 (89.5)	142 (91.6)	0.670^¶^	10 (100)	149 (90.9)	1^¶^
	No	9 (7.7)	6 (10.5)		10 (7.5)	5 (12.5)		2 (10.5)	13 (8.4)		0 (0)	15 (9.1)	
Question 2 Olive oil	≥ 4 spoonfuls/day	51 (43.6)	25 (43.9)	0.973	60 (44.8)	16 (40)	0.593	10 (52.6)	66 (42.6)	0.404	4 (40)	72 (43.9)	1^¶^
	< 4 spoonfuls /day	66 (56.4)	32 (56.1)		74 (55.2)	24 (60)		9 (47.4)	89 (57.4)		6 (60)	92 (56.1)	
Question 3 Vegetables	≥ 2 servings/day	18 (15.4)	6 (10.5)	0.383	22 (16.4)	2 (5)	0.066	3 (15.8)	21 (13.5)	0.730^¶^	1 (10)	23 (14)	1^¶^
	< 2 servings/day	99 (84.6)	51 (89.5)		112 (83.6)	38 (95)		16 (84.2)	134 (86.5)		9 (90)	141 (86)	
Question 4 Fruit	≥ 3 pieces /day	52 (44.4)	21 (36.8)	0.340	56 (41.8)	17 (42.5)	0.936	6 (31.6)	67 (43.2)	0.332	7 (70)	66 (40.2)	0.097^¶^
	< 3 pieces/day	65 (55.6)	36 (63.2)		78 (58.2)	23 (57.5)		13 (68.4)	88 (56.8)		3 (30)	98 (59.8)	
Question 5 Red meal	< once/day	109 (93.2)	48 (84.2)	0.062	124 (92.5)	33 (82.5)	0.072	17 (89.5)	140 (90.3)	1^¶^	10 (100)	147 (89.6)	0.601^¶^
	≥ once/day	8 (6.8)	9 (15.8)		10 (7.5)	7 (17.5)		2 (10.5)	15 (9.7)		0 (0)	17 (10.4)	
Question 6 Butter, margarine or cream	< once/day	101 (86.3)	49 (86)	0.948	115 (85.8)	35 (87.5)	0.787	14 (73.7)	136 (87.7)	0.148^¶^	8 (80)	142 (86.6)	0.630^¶^
	≥ once/day	16 (13.7)	8 (14)		19 (14.2)	5 (12.5)		5 (26.3)	19 (12.3)		2 (20)	22 (13.4)	
Question 7 Sugary or carbonated drinks	< once/day	82 (70.1)	38 (66.7)	0.647	92 (68.7)	28 (70)	0.872	15 (78.9)	105 (67.7)	0.319	9 (90)	111 (67.7)	0.176^¶^
	≥ once/day	35 (29.9)	19 (33.3)		42 (31.3)	12 (30)		4 (21.1)	50 (32.3)		1 (10)	53 (32.3)	
Question 8 Wine	≥ 7 glasses/week	20 (17.1)	8 (14)	0.606	23 (17.2)	5 (12.5)	0.481	2 (10.5)	26 (16.8)	0.484^¶^	1 (10)	27 (16.5)	1^¶^
	< 7 glasses/ week	97 (82.9)	49 (86)		111 (82.8)	35 (87.5)		17 (89.5)	129 (83.2)		9 (90)	137 (83.5)	
Question 9 Legumes	≥ 3 servings/week	28 (23.9)	17 (29.8)	0.405	29 (21.6)	16 (40)	0.020*	7 ()	38 (24.5)	0.271^¶^	3 (30)	42 (25.6)	0.720^¶^
	< 3 servings/week	89 (76.1)	40 (70.2)		105 (78,4)	24 (60)		12 (63.2)	117 (75.5)		7 (70)	122 (74.4)	
Question 10 Fish or seafood	≥ 3 servings/week	30 (25.6)	11 (19.3)	0.355	34 (25.4)	7 (17.5)	0.303	3 (36.8)	38 (24.5)	0.569^¶^	3 (30)	38 (23.2)	0.702^¶^
	< 3 servings/week	87 (74.4)	46 (80.7)		100 (74.6)	33 (82.5)		16 (84.2)	117 (75.5)		7 (70)	126 (76.8)	
Question 11 Non-homemade baking	< 2 servings/week	72 (61.5)	33 (57.9)	0.654	83 (61.9)	22 (55)	0.431	11 (57.9)	94 (60.6)	0.817	8 (80)	97 (59.1)	0.319^¶^
	≥ 2 servings/week	45 (38.5)	24 (42.1)		51 (38.1)	18 (45)		8 (42.1)	61 (39.4)		2 (20)	67 (40.9)	
Question 12 Nuts	≥ 3 servings/week	46 (39.3)	16 (28.1)	0.146	51 (38.1)	11 (27.5)	0.221	9 (47.4)	53 (34.2)	0.258	4 (40)	58 (35.4)	0.746^¶^
	< 3 servings/week	71 (60.7)	41 (71.9)		83 (61.9)	29 (72.5)		10 (52.6)	102 (65.8)		6 (60)	106 (64.6)	
Question 13 White meat preferably	Yes	88 (75.2)	47 (82.5)	0.282	99 (73.9)	36 (90)	0.032*	14 (73.7)	121 (78.1)	0.771^¶^	7 (70)	128 (78)	0.695^¶^
	No	29 (24.8)	10 (17.5)		35 (26.1)	4 (10)		5 (26.3)	34 (21.9)		3 (30)	36 (22)	
Question 14 Sautéed dishes	≥ twice/week	40 (34.2)	11 (19.3)	0.043*	41 (30.6)	10 (25)	0.495	11 (57.9)	40 (25.8)	0.004*	7 (70)	44 (26.8)	0.007^¶^*
	< twice/week	77 (65.8)	46 (80.7)		93 (69.4)	30 (75)		8 (42.1)	115 (74.2)		3 (30)	120 (73.2)	

*MEDAS* Mediterranean diet adherence screener

Chi square test and ^¶^Fisher’s exact test

^*^p < 0.05 statistically significant

The multivariate analysis of all the sensitivities considered (to pressure, vibration and thermal) showed that only sensitivity to pressure (assessed with the monofilament) was significantly related to adherence to the MD (OR = 2.9, 95%CI 1.02–8.22; *p* = 0.045). Nagelkerke R^2^ model 0.83 (see Table 4).

**Table 4:** Multivariate analysis to adherence to the MD

Type of sensitivity	B	Standard error	Wald	gl	Sig	Exp (B)	95% CI for EXP (B)	
							Lower	Upper
Thermal sensitivity	0.601	0.551	1.188	1	0.276	1.824	0.619	5.376
Pressure sensitivity	1.049	0.525	3.990	1	0.046*	2.855	1.020	7.993
Vibration sensitivity	0.965	0.691	1.948	1	0.163	2.624	0.677	10.175
Constant	− 2.837	1.736	2.671	1	0.102	0.059		

## Discussion

Only 19% of the patients in the study population adhered to the MD. The results obtained in the present study were compared with those of previous research in which the MEDAS-14 was also used and which applied the same criteria for MD adherence classification (score ≥ 9). In our study, the mean value obtained for adherence to the MD was slightly higher than the 12% obtained for the Spanish adult population by León Muñoz et al. [35]. However, other studies have reported higher values in this respect [37–39]. In the research by Zaragoza-Martí et al. [36], around half of the elderly subjects (51.7%) presented adherence to the MD. This study sample was composed mostly of women (81.8%), and the vast majority (96.2%) had an active lifestyle. These circumstances might have biased the results obtained. Finally, the adherence of our patients to the MD was much lower than that reported in a study of patients with heart failure [37] or ischaemic heart disease [38] in which cases the adherence to the MD was acceptable (58.9% and 63%, respectively). On the other hand, the first of these was a pilot study without sample size calculation, and so its results could lack external validity.

Other investigations have studied adherence to the MD in various populations, also using the MEDAS-14, but with a different classification system [11, 26, 39–41].

We believe it important to identify the areas of the MD in which our study population presented poor compliance with the recommendations. These items include the consumption of vegetables, wine, fish, legumes, stir-fried vegetables, nuts, fruit and olive oil. However, patients with certain pathologies are advised to restrict the consumption of some foodstuffs: this is the case of alcoholic drinks for patients with arterial hypertension; and that of oil, legumes and nuts for persons with obesity. This circumstance could have influenced the results obtained for compliance with the MD.

Indeed, the low level of consumption of vegetables, legumes and wine observed in the present study coincides with previous findings in this respect [35, 37, 38, 41, 42]. Moreover, Azorín-Ros et al. [41], in addition to finding insufficient consumption of the above-named dietary components, also reported poor compliance (47.3%) with the recommended limit for the consumption of red meat and cured meats (less than once a day). In the study by León-Muñoz et al. [35], the preferential consumption of white meat was another area in which the study population, overall, fell short of the MEDAS recommendations (24%). Finally, Álvarez-Fernández et al. [42] reported that the consumption of stir-fried vegetables was one area in which compliance was very good; this finding was in contrast to our own.

Ghaemi et al. [22] observed a significantly lower incidence of neuropathy in patients with T2DM who adhered to the MD (*p* < 0.001). Furthermore, in a study conducted by Smith et al. [43], it was observed that a lifestyle intervention involving dietary and exercise counseling in patients with neuropathy associated with glucose intolerance resulted in partial cutaneous reinnervation and pain improvement. These results may corroborate those found in the present study, which is the first to separately assess the various parameters that may be affected in DPN among patients with T2DM. In this respect, where we demonstrated that patients with altered sensitivity to pressure and vibration obtained lower scores on the MEDAS-14. However, multivariate analysis revealed significant associations only with adherence to the MD and with altered sensitivity to pressure, although the latter test is the most commonly used to diagnose DPN and is recommended by all international guidelines. The monofilament test of pressure sensitivity is currently the best means of screening for neuropathy [27]. Furthermore, the altered pressure sensitivity revealed by the monofilament test is a risk factor for the development of ulcers and/or amputation of the lower limb. However, our results differ from those found in the randomized controlled trial conducted by Kender et al. [44] demonstrated a significant decrease in the motor nerve conduction velocity of the tibial nerve, as well as a reduction in the heat pain threshold in patients who adhered to the MD. However, it’s important to note that these patients only followed this diet for 5 days per month over a total period of 6 months.

According to our bivariate analysis, the women in the study population presented greater adherence to the MD and recorded a higher score on the MEDAS-14 (*p* < 0.001; *p* = 0.003). These findings are in line with those obtained by Theodoridis et al. [45] and Rodríguez-Caldero [26] (*p* = 0.016 and *p* = 0.0048, respectively). However, they differ from the results obtained by Álvarez-Fernández et al. [42], for whom men scored more highly than women on the MEDAS-14 (7.8 and 7.5 points, respectively; *p* < 0.001). Nevertheless, although the male patients presented greater adherence to the MD (38.5% and 31.7%, respectively), the difference was not statistically significant. Similar findings were reported by León-Muñoz et al. [35]

The results of our study reflect a significant association between the MEDAS-14 score and BMI (*p* = 0.047), with normal weight patients obtaining the highest scores on the dietary questionnaire. These results are similar to those achieved by Zaragoza-Marti et al. [36], in that low adherence to the MD was associated with higher rates of obesity (*p* = 0.178), an increased waist-hip ratio (*p* = 0.81) and higher body fat percentage (*p* = 0.010). In the study by Kalkuz et al. [40] BMI (*p* = 0.111), fat proportion (*p* = 0.054), body fat mass (*p* = 0.024) and lean body mass (*p* = 0.114) were all higher in patients who did not adhere to the MD. Accordingly, this diet is generally recommended to patients seeking to lose weight and prevent obesity [46, 47]. However, the latter results differ from those reported by Ghaemi et al. [22], in which there was an inverse association between compliance with the MD and obesity (*p* < 0.001) in patients with T2DM.

It is noteworthy that overweight patients scored lower on the MEDAS-14 questionnaire compared to obese individuals. This could be attributed to the specific distribution of body fat, as the preferential accumulation of fat in the lower body, characteristic of overweight patients, has been associated with a more favorable risk profile compared to the expansion of adipose tissue in the abdominal region. These findings underscore the importance of considering fat distribution when assessing adherence to the MD and potential associated risks in our studied population [24].

Obesity, dyslipidemia, and glucose impairment are often comorbidities that contribute to DPN in individuals with metabolic syndrome. Approximately 10–40% of individuals with obesity exhibit neuropathy with involvement of small and medium-sized nerve fibers [48]. In our study, we did not find a significant association between obesity and DPN, perhaps due to the lack of consideration for fat distribution using abdominal circumference measurement. However, it is plausible that adipose tissue expansion could impact the progression of DPN symptoms in patients with T2DM. Nevertheless, we did identify a relationship with dyslipidemia as a potential risk factor [49, 50]. Therefore, controlling dyslipidemia is of utmost importance, as it can have a substantial impact on symptom improvement and the prevention of additional complications, such as foot ulcers or infections.

In our research, a significant relationship was observed between physical exercise and the MD (*p* = 0.003). Similarly, Azorín-Ros et al. [41] concluded that hypertensive patients who were sedentary for less than two hours a day had greater adherence to the MD (*p* = 0.025), and León-Muñoz et al. [35] concluded that the MD was associated directly with physical activity (*p* = 0.001) and inversely with time spent watching television (*p* < 0.001). However, among patients not adhering to the MD, there was a higher prevalence of physically active individuals compared to those maintaining a sedentary lifestyle. This discrepancy may be attributed to the possibility that patients with poor metabolic control due to an inadequate diet are engaging in higher levels of physical exercise, following medical recommendations, with the aim of improving their health condition.

Furthermore, our results indicate that adherence to the MD was higher among the patients who had had T2DM for less than 10 years (*p* = 0.035). This finding is in accordance with Ghaemi et al. [22], according to whom the duration of T2DM was higher among the patients who did not consume the MD (*p* = 0.01).

MD consumption was significantly associated with a higher educational level (*p* < 0.001). These results coincide with those found by León-Muñoz et al. [35], who identified an inverse socioeconomic gradient with healthy eating (*p* < 0.001). Furthermore, Álvarez-Fernández et al. [42] and Azorín-Ros et al [41] reported that adherence to the MD was stronger among people in permanent employment (*p* < 0.001) or who belonged to a higher social class (*p* = 0.008), respectively.

The present study is subject to some limitations. The first is that of the non-probabilistic, consecutive sampling technique used. However, this is the method most commonly employed in observational studies and there is nothing to suggest that the characteristics of the patients recruited to our study differ significantly from those of other patients who meet the selection criteria.

Another limitation of this study is that adherence to the MD was evaluated by means of a self-completed questionnaire; in other words, each patient assessed and reported their own food consumption, without oversight or control by the researcher. Furthermore, the existence of recall bias cannot be ruled out when food consumption is assessed retrospectively. However, the MEDAS-14 has been validated previously [47]. Another consideration is that the seasonality of foods should be considered, as this factor could lead to differences in consumption patterns. It could also be interesting to record the duration of your eating pattern or habit and whether there have been any changes in it.

Finally, this study presents the limitations typical of a cross-sectional study. Thus, we were unable to establish a cause-effect relationship nor could we determine whether the declared dietary habits referred to long-term consumption habits or were the result of a recent change. In view of these considerations, our study hypothesis should be further investigated in future prospective studies.

In conclusion, the results obtained in this study highlight the significant importance of the MD in the prevention and mitigation of neuropathy complications in patients with T2DM, by attenuating associated risk factors such as inflammation and vascular dysfunction. The MD, abundant in antioxidants and healthy fats, has demonstrated beneficial effects on cardiovascular health and systemic inflammation, both contributors to DPN. Accordingly, it is essential that health professionals and patients alike recognise the importance of the MD and work together to promote its uptake and adherence. These findings support the need for future research to delve into the underlying mechanisms and clinical impact of adherence to the MD in DPN.

## Data Availability

There is no data repository available.
